# Self-Care Experiences of Adults with Chronic Disease in Indonesia: An Integrative Review

**DOI:** 10.1155/2020/1379547

**Published:** 2020-08-25

**Authors:** Nurul Akidah Lukman, Annette Leibing, Lisa Merry

**Affiliations:** Faculty of Nursing, University of Montreal, Canada H3T 1A8

## Abstract

We conducted a literature review to document what is known regarding the self-care experiences and various influencing factors among adults living with chronic disease in Indonesia, from the perspective of those living with the illness. We searched CINAHL and Google Scholar to identify peer-reviewed research focused on men and/or women living with a chronic disease (the most prevalent) in urban or rural settings in Indonesia. Using a “Self-Care of Chronic Illness” framework as a guide, information on self-care experiences and how various factors influence these experiences, was extracted and synthesized. Nine studies were included (3 quantitative; 6 qualitative). Self-care involves maintaining well-being through different strategies (e.g., foot hygiene, seeking information/care, praying, diet, resting, and simplifying life), following prescribed treatments, and using traditional remedies. Religion sometimes serves as a means for taking care of one's health (e.g., prayer), or as a source of motivation to self-care, while in other instances, it results in a fatalistic attitude. Which treatments (conventional versus traditional) are sought, it is affected by an understanding of the disease and treatments, which is shaped by beliefs, values, emotions, health literacy, and SES. The literature shows that family, especially women, has a key role in providing support. Community organizations also play an important supportive role, particularly for patients in rural areas. Significant barriers to healthcare include costs and care not being well-adapted to the psychosocial needs and contexts of patients. The literature highlights a disconnection between the self-care experiences and how healthcare and support are delivered. To better support self-care, healthcare professionals should use a personalized approach; however, more research is needed to gain a better understanding of what patients want and expect regarding how religion, beliefs, life circumstances, and the use of alternative therapies should be addressed within the patient-professional dynamic.

## 1. Introduction

Rising morbidity rates and a high prevalence of death due to chronic disease, including cardiovascular disorders (35%), respiratory illnesses (6%), and diabetes (6%), are of growing concern in Indonesia [1]. For those living with a chronic disease, it has a significant impact on functioning, quality of life, and well-being, while for society, it has implications for productivity and healthcare costs. Although prevention and health promotion initiatives are needed to curb the rising rates of chronic disease, there is also a need to improve healthcare and services in order to better support those already affected by an illness [2].

Self-care is an essential aspect of chronic disease management [3]. It involves the actions and behaviors implemented by an individual towards monitoring and managing their chronic illness and maintaining health [4]. Self-care experiences are complex as individuals may have different perspectives on their illness, and their priorities, expectations for support, and how they go about carrying-out their self-care may vary. These experiences are also shaped by several factors, including cultural beliefs and values, biomedical knowledge/health literacy, confidence and skill, support from others, and access to care [4]. Indonesia's population is ethnically diverse; there are over 300 ethnic groups, each with their own culture and convictions [5, 6], and these influence health beliefs and practices [6], including the use of complementary/alternative medicine [7–10]. Indonesians are also known for their religiosity, and for many, faith and spirituality have an important role in how they make sense of and face an illness [10–12]. Education levels also vary among Indonesians [13], and this affects health literacy, which in turn also has an effect on health behaviors and treatment choices [7–12, 14–16]. Culturally, Indonesians tend to be collective and family-oriented [15, 17] and have more traditional views on gender roles, which shape expectations regarding support and assistance for when one is sick or suffering [7, 9, 14, 15]. Additionally, access to healthcare and support services may differ depending on where one lives and on their socioeconomic status (SES) [7–10, 12, 16]. To adequately care for and promote self-care, it is therefore imperative for policymakers and healthcare providers in Indonesia to understand the self-care experiences of those living with a chronic illness and how various factors may influence these experiences.

The aim of this review was to document what is known regarding the self-care experiences and various influencing factors among adults living with chronic illness in Indonesia, from the perspective of those living with the illness.

## 2. Materials and Methods

We used an integrative approach and aimed to include a mix of empirical research in the review [18]. We used the “Middle Range Theory of Self-care of Chronic Illness” by Riegel et al. [4] as a framework. In this theory, self-care is defined as the actions and behaviors implemented by an individual towards monitoring and managing their chronic illness and maintaining health [4]. *Self-care maintenance* refers to the behaviors performed by patients to sustain general well-being and to preserve health physically, emotionally, socially, and spiritually [4]. For those with chronic illness, this often includes lifestyle practices such as preparing healthy food, coping with stress, exercising, and maintaining social activities [4]. *Self-care monitoring* refers to the routines and actions that chronic illness patients use to assess the illness (e.g., checking blood glucose levels in patients with diabetes and weight checks in patients with heart failure) so that interventions may then be implemented towards controlling symptoms and/or improving outcomes [4]. *Self-care management* is the active implementation of the treatments and taking medications to manage the disease and prevent health deterioration [4]. Although the specifics of monitoring, maintenance, and management vary, all of these components are part of the self-care experience irrespective of the illness.

According to theory by Riegel et al., a number of factors influence self-care experiences including one's skill level and motivation, functional and cognitive (biomedical knowledge and health literacy) abilities, cultural beliefs and values, lifestyle habits, support from others, and access to care. Some of these are particularly relevant to adults' self-care experiences in Indonesia, including culture and values, biomedical knowledge/health literacy, support from others, and access to care. *Culture and values*, including *religious beliefs*, affect attitudes and perceptions of health and disease, including their causes, and how one should respond to and treat the illness. *Culture* also influences lifestyle choices, which can be essential in maintaining health when living with a chronic disease [4]. *Biomedical knowledge* and *literacy* levels are influenced by education (formal and informal) and will affect how an illness, symptoms, and treatments are understood, while *support from family*, *friends*, *community*, and *healthcare professionals* (*HCPs*) can be a key in helping patients enact self-care, for example, decision-making regarding treatment choices, providing emotional and practical support [4]. Finally, *access to healthcare services* is a significant factor in self-care as it influences which treatments, including medicines and lifestyle recommendations, are followed [4]. Each of these factors (support, access to care, biomedical knowledge/health literacy, and beliefs), as well as the others mentioned in the theory (e.g., motivation and skill), are overlapping and have an influence on one another.

### 2.1. Search Strategy

We searched the CINAHL database. Subject headings and keywords used related to self-care (e.g., self-care, self-management, self-efficacy, and long-term care), chronic disease (e.g., chronic disease, chronic illness, long-term disease, hypertension, high blood pressure, diabetes mellitus type 2, type 2 diabetes mellitus, cardiovascular disease, heart failure, chronic kidney disease, end-stage renal disease, osteoarthritis, arthritis, hemodialysis, and dialysis), and Indonesia (Indonesia, Indonesian, and Indonesian patients). No language or year limitations were applied. The search was conducted during the month of May 2019. The detailed search strategy is presented in Table 1.

Additional studies were also identified by perusing the reference lists of included literature and searching for “related articles” via Google Scholar.

### 2.2. Inclusion and Exclusion Criteria

The inclusion and exclusion criteria are summarized in Table 2. To be included, studies must have described or examined the self-care experiences of adults (men and/or women) living with a chronic illness in Indonesia, including rural and urban areas. Studies that examined self-care experiences of people with chronic illness across several countries (e.g., low-middle-income countries and south-east Asian countries) without any specific mention regarding Indonesia were excluded (i.e., results for Indonesians had to be reported separately). The literature was also restricted to studies that focused on the most prevalent chronic diseases in Indonesia (i.e., hypertension, cardiovascular diseases, chronic kidney diseases, type 2 diabetes mellitus, arthritis, and chronic obstructive pulmonary disease) [1]. Studies that focused on people with cancer were excluded since cancer presents unique challenges and because it is also considered an acute illness that involves intensive treatments and hospitalization. We also excluded pregnant women living with a chronic illness, since pregnancy is a distinct life event and a time when women are experiencing a range of physical, emotional, and social changes that also require care and support. Since the objective was to understand self-care experiences from the point of view of those living with the illness, we excluded studies that examined self-care from the perspective of healthcare professionals. We only considered peer-reviewed research, including quantitative, qualitative, and mixed methods' studies published in English and *Bahasa*.

All citations were downloaded and managed using Endnote X9 software. NAL was responsible for screening and selecting the literature. This process consisted of three steps: (1) a review of titles in order to eliminate duplicates and records that clearly did not meet the inclusion/exclusion criteria, (2) a review of abstracts to identify potentially relevant papers, and (3) a review of full-texts to confirm eligibility.

### 2.3. Data Extraction, Collation, and Reporting

For all articles meeting the inclusion criteria, we extracted the following data: (1) paper characteristics (i.e., authors and publication year) and (2) study information, including the objective, research design, population (e.g., age group, education level, and socioeconomic status) and chronic illnesses (e.g., hypertension) studied, research location (city/region, urban vs. rural), data collection methods, and key findings/themes. We also extracted information about self-care experiences and how the various factors influenced these experiences. We used the Riegel et al. [4] framework as a guide for this process. NAL read each article in full and extracted the information into tables in a word document. LM and AL supervised and supported NAL through ongoing discussions; LM also reviewed three papers to confirm the quality and completeness of the data extracted. The paper characteristics and study information were summarized into tables. The information on self-care experiences and the influencing factors were synthesized into a narrative text and organized under the following headings: religion; biomedical knowledge and health literacy; cultural expectations; support from family, peer, HCPs, and community; and barriers to care. All authors contributed to the synthesis and writing of the results.

## 3. Results

**Figure 1:**
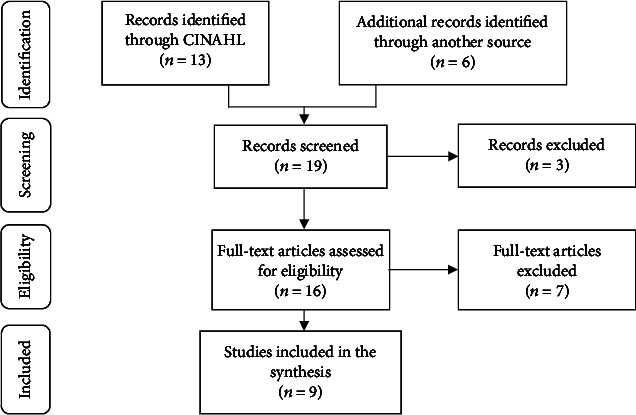
Identification and selection of the literature.

Figure 1 presents the results from the literature search. Nineteen records were identified (13 through the database searches, 1 through the review of reference lists, and 5 through Google Scholar); three were excluded because they were conference abstracts/proceedings. Sixteen full-text papers were then assessed for eligibility and seven articles were subsequently eliminated because they did not meet the inclusion criteria (i.e., participants did not have a chronic illness, had cancer or were pregnant, or the study focused on the perspectives of healthcare professionals). Nine papers (English publications), including six qualitative and three quantitative studies, were selected for the integrative synthesis. A summary of the included literature is presented in Table 3.

Table 4 summarizes the characteristics of the included papers. All nine were published within the last 10 years, and most were published in 2018 and 2019. Studies were conducted in a mix of urban and rural settings, and the participants were mostly female (*n* = 7), elderly (*n* = 5), low educated (*n* = 5), and living with diabetes (*n* = 4) or hypertension (*n* = 3). Regarding the factors influencing self-care, all of the studies considered access to care and biomedical knowledge, health literacy, and/or culture (*n* = 9), while peer support was only mentioned in two papers.

### 3.1. Chronic Illness Self-Care Experiences and Influencing Factors

#### 3.1.1. Religion

Research on the self-care experiences of Indonesians living with chronic illness shows that religion and faith can have a significant influence on experiences, both positively and negatively [10–12].

The positive effects of religion are evident across Indonesians with different illnesses and living in various regions and settings. For example, in the study with Muslim Indonesians living with end-stage renal disease (ESRD) undergoing hemodialysis in urban regions of Pekanbaru, a male participant mentioned that prayer gave him spirit and serenity and helped him sleep [10]. Going to the mosque or listening to the radio online to hear Islamic preaching also motivated some participants to seek medical treatment and to “not give up” on their illness [10]. Similarly, in the study of Muslim patients with type 2 diabetes mellitus (T2DM) living in urban regions in Yogyakarta, examining the relation between prayer and foot care behavior, results showed a significant association between praying and adopting good foot care behavior; the more patients prayed, the more likely they were to adopt good foot care [11]. The study explained that praying was an occasion, up to five times a day, for patients to wash their feet and therefore it helped them to maintain good hygiene and prevent foot ulcers [11]. The study also explained that patients were more likely to engage in good foot care behaviors because they perceived the illness as God's way of reminding them to take care of their health [11]. It therefore encouraged them to take responsibility for their health and to follow recommended foot care in order to avoid foot ulcers and other complications [11].

In other instances, Indonesians believe that self-care is important but, ultimately, that they have little control over their situation and that it is God who is the one to decide their fate. This fatalistic approach was shown in a study with middle-aged, Muslim women participants living in a rural area, and who had hypertension; these women believed that if they fulfilled their obligations to God, for example, by praying and fasting, that then health would be delivered by God [12]. Their faith was strong, and they believed that everything, including disease and health, was determined by God [12].

#### 3.1.2. Biomedical Knowledge and Health Literacy

Research on the self-care experiences in Indonesia reflects the educational and cultural diversity of the country's population and shows variations in how illnesses and treatments are perceived and understood, and how self-care is practiced [7–12, 14–16].

For some Indonesians, self-efficacy (i.e., confidence and belief in one's ability to achieve a goal), which is associated with high-level education, is an essential and positive contributor to their self-care behavior [9, 12, 14]. In other words, for some patients, a better medical understanding of the disease results in more active and engaged self-care. In two studies, self-efficacy was shown to be positively associated with diet management, taking medications, seeking treatment, and engaging in other prescribed health and care-related activities [9, 14]. Similarly, in another study, one male participant knew that resting would make him feel better and reduce his stress [12]. In the same study, another male participant addressed his stress by going for walks or accompanying his wife shopping. And still in the same study, another participant discussed the importance of controlling his diet and was motivated and integrated this change into his lifestyle. In each of these cases, the patients had biomedical knowledge/health literacy and confidence which helped them enact changes towards improving their health.

In contrast, a patients' lack of health literacy (i.e., capacity to access, understand, and use health information towards making appropriate health decisions) can lead to not taking care of oneself [7, 9]. For example, some patients living with hypertension in rural regions in Yogyakarta did not recognize that a systolic pressure of 150 mmHg wasn't healthy, and since they did not feel any symptoms, they did not seek any medical treatment [9]. Similarly, in another study, some patients believed that their diabetes was “not too bad” if they had boils on their leg [7]. They thought this was only a small wound and not as severe as the foot ulcers of other patients they knew and therefore believed it was not necessary to seek care [7]. Overall, a lack of understanding of the illness and its symptoms prohibited the patients from taking action.

Knowledge about treatments and their effects is also varied and may be shaped by health literacy as well as beliefs and values [7, 9, 12]. For example, in one study, some Indonesians with type 2 diabetes mellitus believed that medications contained chemical materials and that they were dangerous for their body, and therefore, they decided to stop consuming the medication one week after they were prescribed [7]. However, in another study, patients who were aware of their health problems, had an understanding of their medical care, and had had positive experiences in improving their health reported buying medicines, taking prescribed treatments, and seeking medical attention [12]. Similarly, some participants in another study knew that hypertensive medication would bring a positive effect on their body if they took it regularly and therefore they made many efforts to maintain and follow their treatment as indicated.

Understanding the advantages of self-care also seems to be a motivating factor for some Indonesian patients to act towards managing their illness and improving their health [10, 11]. In one study, for example, the participants saw their hemodialysis as an essential therapy to follow for them to feel well and to improve their quality of life [10]. They also recognized that controlling fluid intake, reducing the toxins in their body through diet, resting, and using massage, helped them to manage and control their symptoms including weakness and pain [10]. The positive outcomes therefore stimulated them to further engage in these behaviors [10]. Similarly, in another study with patients living with type 2 diabetes mellitus, when patients believed foot care was a useful treatment, they would perform the foot care behavior [11]. The study explained that patients who believed that their treatment was effective were more motivated to perform better foot care.

In a similar vein, fear of negative outcomes or previous bad outcomes can also stimulate patients to act and better monitor and manage their illness [9, 11, 12]. In one study, some patients with hypertension practiced a healthy lifestyle (e.g., checked their blood pressure regularly, controlled their diet, and exercised) due to their previous experience of having a stroke (a complication of hypertension) [12]. Conversely, the potential adverse consequences and severity of the illness can demotivate patients. For example, when patients in the study by Indrayana et al. felt that their T2DM prognosis was poor, they were less likely to have proper foot care behavior [11]. The study explained that perceiving serious consequences of their illness causes stress and decreases patients' intention and drive to perform foot care. In another study, demotivation in self-care was also noted; however, this was due to the feeling that the medications were not effective [9]. The older patients in the study had tried many treatments and had made many efforts to treat their hypertension, but in the end, felt there was little effect or progress [9]. They therefore preferred to remain untreated as long as they could continue to work and “not feel too dizzy” [9]. This decision was also motivated by the fact that these patients felt that given their older age, they just needed to simplify their life and accept and live with their illness and its outcomes, whatever they may be [9].

Knowledge and where information is sought are influenced by various factors including gender, SES, and personal characteristics. In the study by Dewi et al., in high SES households, both men and women tended to seek health information from various sources, including mass media, magazines, television, and websites, while in low SES homes, women were mostly responsible for the task of finding information and their sources were not as broad [8]. Low SES women did not do research online or by reading, but rather, they tended to seek information from peers or relatives who were living with a similar condition [8]. In the study by Ligita et al., participants reported that they examined and decided what information to use based on their prior knowledge, own experiences, and personal judgment [7]. They also asked for the opinions of others in whom they had confidence (e.g., HCPs, peers, and family) before finally choosing to trust or distrust information [7]. In the same study, some participants also did research before deciding on whether or not to use a treatment (e.g., they searched for information online as they wanted to inform themselves about the function and the side effects of traditional treatments recommended by a friend before choosing to use it or not).

Finally, irrespective of having biomedical knowledge and/or a desire to participate in certain self-care activities (e.g., an active lifestyle), life circumstances can be an important determining factor in actual behavior. For example, men with CVD in the study by Dewi et al. felt that they did not have enough time to exercise [8]. Similarly, in the study by Mizutani et al., some participants living with hypertension hesitated to do exercise because the location of the sports centre was far from their living area [12]. In the same study, other participants wanted to improve their chronic condition by exercising, but they felt that exercising in hot weather would actually worsen their condition [12]. In both studies, participants also believed that their job and farming activities (e.g., planting the fruit, digging, and walking around), respectively, were sufficient as a form of exercise since it was physically demanding [12].

#### 3.1.3. Cultural Expectations

Many beliefs around chronic illness and responses are influenced by culture [8, 11]. The eating behaviors of Indonesians, for example, are often determined by cultural and religious traditions, and these can influence self-care in complex ways. For example, in the study by Indrayana et al., “accepting food and drinks served by another person” was significantly associated with positive foot care behavior [11]. The study explained that participants might have believed that they were not allowed socially or culturally to refuse food served by another person and therefore they compensated by performing better foot care behaviors [11]. Similarly, people with chronic illness may also seek to compensate for the negative effects of smoking [8]. Smoking is a common habit and widely socially accepted in Indonesia, especially among men, and as such is very difficult to quit, even in the face of illness [8]. Compensation is therefore a strategy that may be used to maintain health, rather than to quit the habit [8]. For example, high SES males in the study by Dewi et al. perceived smoking as unhealthy but felt it was needed to help them cope with their job [8]. Therefore, to compensate, they believed that they needed to exercise [8].

Gender roles, which are culturally determined, can also affect how a chronic disease is perceived and self-care is practiced [8]. For example, male patients with cardiovascular disease (CVD) in the study by Dewi et al. believed that they were responsible for earning money to support the family and therefore they needed to prioritize their job and felt that they did not have time to attend to their health [8]. High SES male patients, however, reported that they believed that engaging in a healthy lifestyle could improve their outcomes, but did not have time for these activities and therefore managed and monitored their CVD only by necessary medical examinations and care [8]. Conversely, male patients of low SES believed that their heart disease was their destiny and that they had no control in changing the outcome of their condition [8]. They felt that any activities aimed at prevention would be useless [8].

Culture and beliefs also shape the choice on which treatments and care are used [7, 8]. For example, in Ligita et al., some patients with T2DM living in Pontianak (the capital of the Indonesian province of West Kalimantan) preferred to use unconventional therapies, as they believed that taking medications and drinking less water, as were recommended by healthcare professionals (HCPs), would exacerbate their illness [7]. Instead of prescribed medications or treatments, these patients therefore used traditional medicines and other treatments, for example, a magnet device around their waist [7], which is believed by some people to heal diabetic neuropathy by increasing the blood flow on their lower extremities and reducing their blood glucose levels [19]. Similarly, in the study by Dewi et al., Javanese patients living in Yogyakarta perceived that “too much exposure to wind” was what contributed to them developing cardiovascular disease (CVD); they also referred to CVD as “angin duduk” (sitting wind sickness) [8]. They also believed that their “angin duduk” could only be cured by rubbing the back with traditional oil and then scratching it with the coin [8].

For some, herbal medicine is used as the core treatment for their illness and medical treatment is considered the last resort [7, 9]. In these cases, the use of alternative medicines lowers patients' intentions and motivation to seek medical treatment and leads them to visit doctors only when their condition is severe [7, 9]. For example, in the study by Rahmawati and Bajorek, some patients preferred to treat their hypertension by consuming certain foods including cucumbers, melons, watermelon juice, grated carrots, and said that they only visited medical services when they felt natural approaches had no effect [9]. Similarly, in a study of patients with T2DM, just following their diagnosis, some sought out traditional medicines and decided to use these instead of medically recommended treatments to manage their glucose levels and treat their blurred vision [7].

In some instances, traditional medicine and healers are believed to be complementary to medical treatment [7, 10]. For example, in the study by Bayhakki et al., some patients with end-stage renal disease (ESRD), while undergoing hemodialysis, also used a traditional treatment called *bedah ayam* (also known as “chicken surgery”; i.e., an alternative treatment that involves a healer slaughtering a chicken and performing rituals with the dead chicken in order to remove the illness from a patient's body) [10]. In the same study, some participants used massage and consumed tamarind extract to relieve pain as it contains antioxidants and has anti-inflammatory effects [20]. Similarly, in the study by Ligita et al., some patients with T2DM also used herbal medication as a complementary treatment to their insulin injections [7].

Some Indonesians decide not to use traditional therapies at all and prefer to only follow medically prescribed treatments [7]. This decision appears to be influenced by a combination of factors, including fear of traditional medicines, support to use prescribed medicine, and beliefs that conventional therapies are more effective [7]. For example, in one study, a female participant did not trust alternative therapies that were suggested by her friend because she feared overdosing since she did not know what amount of the medication would be appropriate for her [7]. Moreover, it was important for her that there be medical evidence showing the effectiveness of the treatment in order for her to feel comfortable taking it [7].

Lastly, for some, traditional approaches may be used initially to manage and treat illness and to maintain health, but then due to adverse effects, are stopped [7–10]. This was evident in the study by Dewi et al., where some patients with CVD who had used traditional methods including rubbing their back with oil and scratching it with a coin reported it had had no effect for them or for other patients with CVD that they knew who had used it [8]. Those with ESRD, in the study by Bayhakki et al., who had used the *bedah ayam*, massage, and tamarind extract, also reverted to only using hemodialysis due to feeling no effect from alternative approaches [10]. In the study by Rahmawati and Bajorek where patients living with hypertension consumed fruit as a primary treatment and natural method for staying healthy, this too was stopped because it had no effect on their blood pressure [9]. Finally, in another study, some participants with T2DM also stopped using herbal medicines because it caused them to be hypoglycaemic [7].

#### 3.1.4. Family Support

Family support includes giving motivation, financial help, and daily life assistance and may be provided by different family members [7, 9, 14, 15]. For example, in the study by Kristianingrum et al., some female participants with T2DM received support from their daughters to iron and wash their clothes, to remind them to consume food, and to take medicine [15]. The women were also accompanied to visit medical services, given money to buy the medications, and encouraged not to do housework and to rest by their sons [15]. Similarly, some older patients in the study by Rahmawati and Bajorek also relied on their children to buy medications and to prepare healthy meals for them [9]. Studies also show that siblings and spouses provide assistance to their ill family members [7, 15]. For example, in two studies, siblings helped by giving reminders to monitor blood sugar levels, by accompanying their brothers/sisters to visit medical services, and by providing support to follow recommended diets [7, 15]. Lastly, some women in the study by Kristianingrum et al. were helped by their husbands to prepare meals and to take a bath [15].

Family can also be a source of intrinsic motivation for self-care by providing a sense of purpose [10]. In one study, some participants reported that they underwent hemodialysis because they wanted to be well and survive their illness so that they could be around to see their children's success [10]. They also felt that their families needed them, and this therefore inspired them to stay healthy [10].

Family support in some instances is gendered. For some Indonesian men, they consider that their family members, especially women, are responsible for assisting with their self-care [8, 10, 12, 15]. For example, in the study by Bayhakki et al., some men with ESRD asked their wives to drive, prepare meals, and help them go to the restroom [10]. Similarly, in the study by Kristianingrum et al. with Indonesians with T2DM living in urban and rural regions in East Java, male participants also relied on women to help them for a bath, to cook for them, and to remind them to control their stress and eat healthy food [15]. Also, in another study, some of the male participants relied on their wives to remind them not to work so hard [12]. In Dewi et al., women also reported that they felt it was their responsibility to care for their sick family members [8].

Support from family can have both positive and negative effects on self-care [7, 11]. In the study by Amelia et al., motivation from the family, including encouragement and support to eat healthy food and nutrient intake, was positively associated with patients' self-care behavior [11]. Positive feedback helped patients maintain a healthy diet and to control their blood glucose levels [11]. Also, in the same study, money provided from family to cover regular medical examinations was also associated with patients seeking and accessing medical care [11]. Conversely, in another study, a participant reported that they had stopped using insulin to treat their diabetes, after being advised by their older sister that they would become dependent on the medication [7]. Out of respect for the sister and the belief that they had been given good advice, the participant had followed his sister's recommendation [7].

Lastly, some patients, especially older adults, hesitate to ask for help/support from their family, unless they are in an urgent situation [9]. For example, in the study by Rahmawati and Bajorek, some older participants did not want to bother their children when they felt a headache or dizzy [9]. They preferred to self-care independently without any help [9]. They would, however, ask their children to help to accompany them to visit the “village nurse” when they felt very ill [9].

#### 3.1.5. Peer Support

Fellow patient support also seems to be a key source of support for some Indonesians living with chronic illness in Indonesia [7, 12]. For example, in one study, some patients mentioned that sharing experiences with other patients living with similar conditions including information about symptoms and treatment was helpful for them in dealing with their illness [7]. Similarly, in another study, some of the male patients stopped smoking on the advice of friends who also had the same disease and who had been advised by their doctors to stop smoking in order to lower their blood pressure [12].

#### 3.1.6. Support from Healthcare Professionals

Healthcare professionals (HCPs) seem to mainly provide informational support. For example, in the study by Amelia et al., which analyzed factors affecting self-care behavior of patients with T2DM, it was found that information provided about the development, prevention, and treatment of illness by physicians was significantly associated with positive self-care behavior, including controlling blood sugar levels and engaging in other activities to generally maintain health [14]. Similarly, in the study by Ligita et al., some participants reported that HCPs provided them information regarding the treatment and the effects of taking medication regularly versus irregularly, and they used this knowledge to prevent complications of their diabetes [7]. In two other studies conducted in rural areas in Indonesia, patients with hypertension reported obtaining information about reducing salty food and sweets [9, 12]; while in the study by Rahmawati and Bajorek, some patients received information about physical activity from the village midwife, as ways to help manage their diseases.

Informational support from HCPs is not always helpful. This is largely due to information not being well-communicated and/or poorly understood by the patient. This was evident in the study by Ligita et al., where there was a communication breakdown between the HCPs and the patient, and so the patient came to his own conclusions about how best to manage his diabetes. His understanding was that as long as he took the medications, he could eat whatever he wanted [7].

#### 3.1.7. Community Support

Support from the community has also been shown to be essential in the self-care experiences of Indonesians [8, 10, 12]. This includes support from neighbors, the religious community, and community organizations [8, 10, 12]. The latter also includes care and support from lay healthcare workers.

In the study by Bayhakki et al., some Muslims with ESRD mentioned that they were visited by members of their mosque and this enhanced their spirit and motivated them to take better care of themselves [10]. Neighbors and *Kader* (i.e., a health volunteer worker in the community who visits patients and provides care at home) can also be helpful in patients' self-care [12]. In the study by Mizutani et al., some women patients reported being helped by their neighbors, such as escorting them to medical appointments, and were also reminded or accompanied by the *Kader* to visit community support services [12]. In some cases, *Kader* also provides direct care (e.g., blood pressure check and giving health information) [12].

In the study by Dewi et al., participants received support from the *Pemberdayaan Kesejahteraan Keluarga* (*PKK*)—an influential women's group that encourages community participation towards improving family welfare—and *Pos Bina Terpadu Penyakit Tidak Menular* (*Posbindu PTM*)—a women's group that supports the health of people living with noncommunicable diseases (NCD). These are two well-known organizations in Indonesia that are located in the community and provide a range of support care, including medical examinations (e.g., blood pressure and cholesterol level measurement/checks), and health education to patients—these services are predominantly delivered by lay healthcare workers (i.e., individuals with little training and formal healthcare education) [8, 12]. These organizations are therefore key community supports for many Indonesians with chronic illness as they are helpful to patients by providing direct support to aid them in monitoring and managing their illnesses [8].

Both gender and SES are essential factors in community support. In the study by Dewi et al., it was women of low SES who were responsible for supporting health at the community level through community organizations, such as the *PKK* and *Posbindu PTM*. These organizations were also mainly relied upon by men participants of low SES [8]. For high SES participants, they did not depend as much on the support of PKK and Posbindu PTM and preferred to visit professional medical services instead [8].

#### 3.1.8. Barriers to Care

Barriers to seeking and using care and treatments include cost, fear, or discomfort associated with treatments, location of services, negative experiences, and length/severity of the illness. Cost was an issue in the study by Ligita et al., where participants with T2DM hesitated to take medication because they were too expensive [7]. Fear was also an issue in this study where some patients were afraid to do self-injections (of insulin), while some were also worried about the side effects of these injections, including fainting, which could prohibit them from performing their daily activities. Similarly, in the study by Rahmawati and Bajorek, some patients with hypertension also hesitated to seek care because they could not cover the expense [9]. In the same study, some patients also stopped using medications because the smell of the medication made them nauseous or they were tired of taking the medications.

Time and convenience are issues that are usually associated with a patient's employment situation and their sense of responsibility to provide for the family. As already mentioned above, in the study by Mizutani et al. and Dewi et al. with Indonesians living with hypertension and CVD, respectively, male participants positioned themselves as breadwinners who needed to earn money, and therefore, they felt that they did not have time to pursue their health [8, 12]. Their priority was to work and support the family [8, 12].

Challenges associated with the location of healthcare services are mainly when patients live in rural areas. In two studies, several participants hesitated to seek care because the location of healthcare services was far from where they lived and the route to get there was rough terrain and they did not have appropriate vehicles to take these roads [9, 12]. In the study by Rahmawati and Bajorek, for some, especially elderly, walking was also difficult and added to the challenge of getting to health services [9]. For the patients in these studies, it was felt that seeking care in such conditions would actually exacerbate their illness [9, 12].

Generally, patients living in rural areas seem to prefer to access care that is close by and accessible by walking, whether it is community or larger healthcare services [16]. However, services in rural centres often rely on HCPs coming from urban settings, who are sometimes late, and this can be a barrier to care [9]. As shown in Rahmawati and Bajorek, this can create more frustration and results in patients feeling disappointed and losing trust in the care-providers and services and therefore further lead to patients not seeking services [9].

Conversely, positive experiences and outcomes when care is sought can stimulate further use of services. For example, in the study by Mizutani et al., several patients with hypertension reported that they regularly checked their condition in *Posbindu PTM* because they felt that the information (e.g., diet recommendations) that was given was very useful for them [12].

Length of time living with an illness is another determining factor on where health services are accessed. In the survey by Rahmawati and Bajorek, participants who were recently diagnosed with hypertension would obtain their care and medication at the community organizations and hospitals nearby (e.g., *Puskesmas*, *Posbindu PTM*, and public hospital) [16]. However, patients who had been living with the disease longer were more likely to obtain their medications in larger hospitals or private pharmacies (e.g., private doctor, private nurse, or private midwife) [16]. The study explained that people with a recent diagnosis perceived that their illness was not severe and therefore did not require more “complex” care or medication, while those who had been living with the disease for a while felt that their condition required more advanced care that could only be provided in private or larger healthcare facilities [16].

Along the same lines, the advanced condition of patients is also sometimes a determining factor on when medical care is sought. This was evident in the study by Bayhakki et al., where participants with ESRD only sought medical treatment if they were no longer able to care for themselves or when the condition was very severe [10]. Similarly, in the study by Rahmawati and Bajorek, some older patients with hypertension reported that they would never visit medical services as long as they felt that they could manage their condition themselves (e.g., take a rest and take available medication) and the symptoms were not bothering their work and/or daily life activities [9]. They felt healthcare services would only be necessary if they felt very ill [9].

Lastly, the expensive cost of medical visits, having a low socioeconomic status, the location of health services, and fear of being given poor prognoses from the HCPs can also contribute to patients' self-medicating rather than using prescription drugs. This was evident in three studies among older Indonesians with hypertension living in rural areas where several participants tended to buy over the counter medications (without prescription) in *Warung* [9, 12, 16], a small family-owned business (e.g., restaurant, café, minimarket) in Indonesia [21], or in pharmacies, to treat their blood pressure [9, 12, 16]. In Rahmawati and Bajorek, patients preferred to take prescribed medication as a treatment to lower their blood pressure only when the location of the health services was not far from their living area [16].

## 4. Discussion

The literature reviewed provides several insights on self-care among Indonesians living with chronic illness in Indonesia including (1) the importance of religion and faith in how one lives with their illness; (2) the variation in responses to an illness that are influenced by a person's background and life experiences; (3) the role of family and community, especially women for self-care support; and (4) the range of barriers that people face in accessing care and services.

The findings of the review highlight how in Indonesia religion may serve as a means for taking care of one's health (e.g., through prayer), or as a source of motivation to partake in self-care. Practicing the Muslim faith provides not only psychological well-being but also physical wellness [10]. This includes, for example, hearing Islamic preaching which can increase one's patience in order to better face illness and be an empowering force to self-care [10], as well as adopting good foot care behavior (i.e., washing the feet), as this is part of the prayer ritual [11]. The role of religion in self-care has also been shown in research conducted in other non-Western countries. For example, a study done in Ghana with people living with end-stage kidney disease, faith was shown to be a driving motivator and method for staying well [22]. Care providers' can thus promote faith and religious practices as a means to support their patients' self-care [10, 22, 23].

In other instances, religion however seems to result in a fatalistic attitude and approach to health. These such attitude and approach to living with an illness have also been observed in other research, where similar to our findings, patients believed that their illness was God's plan and that it was up to God to decide their fate [22]. They also had a strong belief that God would keep them well as long as they fulfilled their obligations to God. “Fulfilling obligations” and being rewarded by God may be interpreted in various ways within and across religious affiliations and thus may have different implications for self-care [23]. For some, they may interpret it to mean that they should solely rely on a reward from God for good health, and not engage in any active disease management or monitoring, whereas others may interpret it to mean that they should actively treat their illness and partake in activities to stay well as part of God's work in addition to their other religious obligations. Care providers should therefore be cognizant of how religion may operate and influence self-care and provide an opportunity to discuss with their patients so that they may understand their patients' beliefs and views and adjust their care accordingly [10, 22, 23].

Our findings also show that Indonesians' responses to living with a chronic illness, including which sort of treatments (conventional versus traditional) are sought, are very much influenced by one's knowledge and understanding of the disease and treatments. In turn, knowledge and understanding are shaped by culture, beliefs, emotions, life experiences, and circumstances and by the level of education/SES and health literacy. Varied responses and behaviors to living with an illness have also been shown in other research conducted in low- and middle-income countries. These studies also found that patients have different views and practices related to diet, exercise, foot care, and which medical and nonmedical treatment they seek as part of their disease management and health promotion activities [22, 24–27]. These results therefore suggest that to best promote self-care, care providers should consider the cultural and social diversity of their patients and adapt their care accordingly. In Indonesia, however, the predominant healthcare model is that HCPs are the experts and that patients should comply with their medical instructions. Currently, there is little collaboration with patients or adaptation to care based on patients' needs or preferences [8]. The results of the review also showed that HCPs mainly provide support in the form of prescribing medications and making recommendations on lifestyle behaviors. Therefore, there seems to be a disconnection between the realities of patients, their self-care experiences, and how healthcare is delivered.

Regarding support, the literature reviewed showed that family, especially women, has a key role in supporting sick family members including direct assistance with self-care as well as emotional encouragement. This is consistent with what is known regarding the role women tend to play in family life in Indonesia. In addition, in Indonesian culture, caring for family members is perceived as an obligation and a form of adhering to religion [15]. Similar to our findings, other research has shown that family is often closely involved and may provide a range of support and care to their sick loved ones, especially in low-resource countries/areas [22, 26, 27]. This research also highlights that family support can be positive as well present challenges, for example, causing tension between the ill family member and their family caregivers regarding how to stay healthy and manage the illness [22, 26, 27]. Given the extensive involvement of family, in particular, female family members, family should not only be included in the care process of the ill family member but also support should be made available to them in order to ensure that they do not experience caregiver burnout. This is especially so in the context where women may not communicate their challenges as they are culturally expected to care for their ill family and where women's roles in Indonesia are expanding and more and more women are entering the workforce (i.e., take more roles outside the home) in order to help the family economically, which is increasing their overall workload [28].

In Indonesia, women also seem to play a pivotal role in supporting self-care via their involvement in community organizations (i.e., *PKK* and *Posbindu PTM)*. The literature suggests that community organizations are essential resources for ill patients, particularly for low-SES and those living in rural areas where medical services are less available. As the burden of chronic disease increases, these community services will need to be strengthened, not only to safeguard against these resources being depleted (and women taking on further responsibilities) but also to ensure that health inequities between low and high-SES patients are not exacerbated. Currently, there are fewer HCPs working in rural areas compared to urban centres. Moreover, given the geographical distance of the rural service centres from the cities, laywomen as well as *Kader* provide services with little supervision or input from professionals [29]. Consequently, care tends to be suboptimal and largely focused on just providing medication; little attention is given to prevention and health promotion [29]. Therefore, more education, training, and supervision of laywomen and *Kader* are needed to improve the quality of service delivered in smaller, rural communities [29]. Supervision may be enhanced by improving (virtual) collaborations between community services and medical education institutions [29]. Increasing healthcare human resources, especially the number of nurses, and implementing strategies to improve the distribution of HCPs across the country (e.g., salary incentives, paid housing, and professional development opportunities) are also required [30].

Lastly, the research shows that some Indonesians face barriers in trying to use health information and services, including the cost, time and inconvenience, the location of services, and emotions and fears around care. Cost and location of services are known access barriers and are not unique to Indonesia [22, 24, 26, 27]. However, given the significant social and economic inequities that exist in Indonesia, the negative effects of these barriers are amplified, especially in remote areas where they are more common. Since 2014, in an effort to try and overcome cost issues, the government introduced *BPJS* (*Badan Penyelenggara Jaminan Sosial*), a free universal health coverage. However, only patients living in urban areas benefited since rural areas lacked the infrastructure and care personnel to deliver services. This further reinforces the need to implement strategies towards making healthcare more widely available across the country. Moreover, since health-seeking behavior and use of services are shaped by emotions and fears, which are influenced by personal beliefs, education levels, previous experiences with the healthcare system, and how advanced their illness is, this further emphasizes that lay care-providers and HCPs should be giving psychosocial support and doing more to personalize care to promote the use of services. The healthcare system, including community services, also need to be better adapted to the life circumstances of patients. The literature however reveals little about what patients expect and would like exactly from healthcare professionals, including how religion, beliefs, life circumstances, and the use of alternative therapies should be addressed and managed within this patient-professional dynamic. Future research in this area is warranted.

### 4.1. Implications for Care and Policy

Given the need for more personalized care, implementation of a person-centred care (PCC), an established healthcare approach used in many high-income countries (e.g., UK, USA, and Australia), may have relevance in Indonesia [6]. In PCC, healthcare providers deliver care that considers a patient's values and characteristics and centres on the patient's personal experience. Self-efficacy is also a central concept to this approach whereby interventions aim to enhance a patient's level of knowledge, competence, and confidence towards further empowering them to make decisions and to actively participate in being healthy [6]. A PCC approach also involves inviting and including relatives in the care process, which aligns with Indonesian's collective and family-oriented culture. PCC has been shown to improve patient capability to self-care and also their satisfaction in services [6].

There are a number of factors to consider, however, if changes in the approach to healthcare delivery or PCC are to be implemented in Indonesia. Firstly, the decentralization of healthcare has resulted in public health institutions and hospitals becoming more profit-oriented rather than focused on the quality of care. Decentralization was meant for local governments to have more power to adapt and implement healthcare to best suit their populations' needs; however, this has not happened [6]. Instead, there has been poor planning and implementation, and HCPs have been limited in what they can do, and thus, access and quality of care have reduced [6]. Secondly, while a personalized care approach has been introduced through nursing education, nurses unfortunately lack authority and therefore have little influence on how care is delivered [6]. Kader and community personnel have even less clout. In Indonesia, physicians have a more prestigious status and are considered the experts for clinical management [6]. Consequently, nurses have less independence in delivering healthcare and cannot adapt their care to patients' needs [6]. In rural areas, as described previously, the Kader and community personnel are simply not equipped to provide personalized care. In order to implement changes in the healthcare system and to improve access and quality to care, political will, financial investment, and policy changes at all levels of government, national and local, would be needed. The government also needs to do more to elevate the professional status of nurses and to reinforce their identity as health professionals so that patients and society will gain trust in them and they will then be able to function more independently, especially in rural areas where fewer services are available [6]. To this end, nurses need more standardized training and education to improve their knowledge, skills, and capacity in delivering interventions. To further promote personalized care (or PCC) across the healthcare system, it also needs to be further integrated into medical school so that physicians are also more likely to adopt and endorse this approach.

### 4.2. Limitations and Strengths

This review has a number of limitations. Firstly, the search only used one database and only included peer-reviewed publications; grey literature, especially in Bahasa, may have provided useful information that was not captured in journal articles. Secondly, the review focused on the most prevalent chronic illnesses in Indonesia and included only the perspectives of patients; experiences with less common illnesses and the views of HCPs and family caregivers' may have offered different information. Thirdly, male and younger participants were less represented in the studies, providing less insight about the experiences of these groups. Fourthly, while some studies considered gender, age, SES, and severity/length of time living with the illness as influencing factors, generally, there was a limited examination of these in the studies.

This review, however, does have strengths. Although the number of papers included was small, our approach was inclusive, and the literature covered a broad range of chronic illness self-care experiences across different ethnic and social populations in Indonesia. We also examined each paper in-depth and used the “Middle Range Theory of Self-Care of Chronic Illness” as a guide, in order to produce an extensive, detailed portrayal of self-care experiences and the various influencing factors [4].

### 4.3. Future Research

Future research should explore what patients expect and would like from healthcare professionals regarding how religion, cultural beliefs, life circumstances, and the use of alternative therapies should be addressed in care. Since men and younger people are underrepresented in the review, further understanding of the self-care experiences of these groups is also needed. Furthermore, analysis of how self-care experiences may differ and change over the illness trajectory is also warranted.

## 5. Conclusions

The research shows that Indonesians living with chronic illness experience self-care in a variety of ways and that there is a disconnection between these experiences and how healthcare and support are delivered. The review suggests that to better support self-care, healthcare professionals should use a more personalized approach. More research, however, is needed to gain a better understanding of what patients' want and expect from healthcare professionals to better monitor and manage their illness and to stay healthy.

## Figures and Tables

**Table 1:** CINAHL search strategy.

#	Query	Results
S19	S17 AND S18 AND S16	13
S18	S4 OR S6 OR S7 OR S8 OR S9 OR S10 OR S11 OR S12 OR S13 OR S14 OR S15	704,073
S17	S1 OR S2 OR S3 OR S5	87,054
S16	(MH “Indonesia”)	2,978
S15	(MH “Dialysis+”)	21,117
S14	(MH “Hemodialysis+”)	14,629
S13	(MH “Osteoarthritis+”)	25,399
S12	(MH “Kidney Failure, Chronic+”) OR (MH “Liver Failure+”) OR (MH “Kidney Diseases+”)	79,339
S11	(MH “Kidney Failure, Chronic+”) OR (MH “Renal Insufficiency, Chronic+”) OR (MH “Chronic Disease+”) OR (MH “Kidney Diseases+”) OR (MH “Noncommunicable Diseases”) OR (MH “Kidney, Cystic+”)	131,481
S10	(MH “Heart Failure+”) OR (MH “Myocardial Ischemia+”)	122,998
S9	(MH “Cardiovascular Diseases+”)	521,800
S8	(MH “Diabetes Mellitus, Type 2”)	54,568
S7	(MH “Hypertension+”)	70,017
S6	(MH “Hypertension”) OR (MH “Pulmonary Arterial Hypertension”) OR (MH “Hypertension, White Coat”) OR (MH “Hypertension, Renovascular”) OR (MH “Hypertension, Pulmonary”) OR (MH “Hypertension, Isolated Systolic”) OR (MH “Intra- Abdominal Hypertension”) OR (MH “Persistent Fetal Circulation Syndrome”) OR (MH “Intracranial Hypertension”) OR (MH “Hypertension, Portal”) OR (MH “Hypertension, Renal”)	65,422
S5	(MH “Long Term Care”)	24,508
S4	(MH “Chronic Disease+”) OR (MH “Kidney Failure, Chronic+”)	77,143
S3	(MH “Self-Efficacy”)	19,148
S2	(MH “Self-Management”) OR (MH “Self Care+”)	45,532
S1	(MH “Self Care+”)	45,532

**Table 2:** Inclusion and exclusion criteria.

Inclusion criteria	Exclusion criteria
(1) Studied the self-care experiences of adults (men and/or women) with chronic illness living in Indonesia	(1) Studied self-care experiences from the perspective of a healthcare professional
(2) Focused on the most prevalent chronic diseases among Indonesian adults (i.e., hypertension, cardiovascular diseases, chronic kidney diseases, type 2 diabetes mellitus, arthritis, and chronic obstructive pulmonary disease)	(2) Studied the self-care experiences of individuals living with chronic diseases across a number of countries (e.g., low-middle-income countries and south-east Asian countries) without any specific mention of Indonesia
(3) Peer-reviewed empirical research (qualitative, quantitative, or mixed methods' studies)	(3) Focused on pregnant women living with a chronic illness
(4) Published in English or *Bahasa*	(4) Focused on individuals living with cancer
	(5) Commentaries, theoretical/discussion papers, books, book reviews, editorials, abstracts, conference abstracts/proceedings, and newspaper/magazine articles

**Table 3:** Summary of included literature.

Study	Study objective	Location and setting	Sample	Research design	Methods	Key findings/themes
Amelia et al. [14]. “Analysis of Factors Affecting the Self-Care Behaviours of Diabetes Mellitus Type 2 Patients in Binjai, North Sumatera-Indonesia”	To determine the most dominant factor affecting the self-care behavior of patients with type 2 diabetes mellitus	Binjai, North Sumatera-Indonesia. Urban	115 respondents—male and female, middle-aged adults and elderly, educated and less educated, high and low socioeconomic status (SES)	Descriptive quantitative and explanatory research	Questionnaires	Self-care behaviors of type 2 diabetes mellitus in Binjai were significantly influenced by motivation, self-efficacy, communication, knowledge, and attitude. Motivation was the most dominant factor.
Bayhakki et al. [10]. “Self-Caring in Islamic Culture of Muslim Persons with End-Stage Renal Disease and Hemodialysis: An Ethnographic Study”	To explore self-caring among Islamic persons living with end-stage renal disease undergoing hemodialysis	Pekanbaru, Indonesia. Urban	12 participants—male and female, middle-aged adults and elderly, educated and less educated, high and low SES	Ethnography	Interview, observation, medical records	Identified themes: (1) Meaning of self-caring (2) Actions in self-caring (3) Islamic influences to self-care living (4) Cultural influences to self-care living
Dewi et al. [8]. “Maintaining Balance and Harmony': Javanese Perceptions of Health and Cardiovascular Disease”	To understand patients' perceptions of health and cardiovascular disease	Yogyakarta, Indonesia. Urban	78 informants—male and female, high and low SES	Qualitative description	Focus group discussion, individual interview	Identified themes: (1) The cause of heart disease (2) Men have no time for health (3) Women are caretakers for health (4) Different information seeking pattern (high vs. low SES) (5) The role of community
Indrayana et al. [11]. “Illness Perception as a Predictor of Foot Care Behavior among People with Type 2 Diabetes Mellitus in Indonesia”	To characterize the relationships among demographic factors, foot care knowledge, illness perception, including local beliefs and foot care practices among people with type 2 diabetes mellitus	Yogyakarta, Indonesia. Urban	200 patients—male and female, middle-aged adults and elderly, educated and less educated, high and low SES	Cross-sectional study	Foot care knowledge questionnaire, the brief illness perception questionnaire, the diabetes foot self-care behavior questionnaire, and local beliefs about diabetes mellitus were measured using a developed questionnaire	(1) Knowledge regarding foot care was strongly correlated with foot care behaviors (2) Perception about illness, including the consequences, the timeline, the treatment control, the identity, the concern, and the coherence, was correlated with foot care behaviors (3) The “food-related and spiritual beliefs” factor was related to foot care behaviors (4) The participants who agreed more that “diabetes is only related to food problems; diabetes is a temptation from God; refusing foods and drinks served by another person is impolite” were more likely to have better foot care behaviors
Kristianingrum et al. [15]. “Perceived Family Support among Older Persons in Diabetes Mellitus Self-Management”	To explore perceived family support by older persons in diabetes mellitus self-management	East Java, Indonesia. Urban and rural	9 older people—male and female, educated and less educated	Descriptive phenomenology	Semistructured interview, field notes	Family support included daily activity assistance, help with accessing health services, food preparation, financial support, psychological support, advice, and solutions regarding self-management.
Ligita et al. [7]. “How People Living with Diabetes in Indonesia Learn about Their Disease: A Grounded Theory Study”	To generate a theory explaining the process by which people with diabetes learn about their disease in Indonesia	Pontianak, the capital city of West Kalimantan Province, Indonesia. Urban	28 participants—from inpatient and outpatient settings	Grounded theory	Face to face and telephone interviews	The core category and social process of the theory was *Learning*, *choosing*, *and acting*: *self-management of diabetes in Indonesia*; this process includes five major distinctive categories: seeking and receiving diabetes-related information, processing received information, responding to recommendations, appraising the results, and sharing with others. People with diabetes acted after they had received recommendations that they considered to be trustworthy. Resource issues (affordability and accessibility of therapies) and physiological and psychological reasons influenced peoples' choice of recommendations.
Mizutani et al. [12]. “Model Development of Healthy-Lifestyle Behaviours for Rural Muslim Indonesians with Hypertension: A Qualitative Study”	To explore the perceptions of middle-aged husbands and wives, whose lives were affected directly or indirectly by hypertension, on their healthy-lifestyle behaviors and related reasons for practicing the behaviors	West Java District, Indonesia. Rural	12 married couples	Qualitative description and case study	Semistructured interview	(1) Behaving healthy by eating well, doing physical activity, resting, not smoking, managing stress, seeking health information, seeking healthcare, providing care for family and community, and fulfilling their obligations to God (2) Reasons for practicing healthy-lifestyle behaviors were beliefs, competence, religious support, prior experience, social support, and health system support (3) Reasons for not practicing healthy-lifestyle behaviors were personal, social, and environmental barriers
Rahmawati and Bajorek [9]. “Understanding Untreated Hypertension from Patients' Point of View: A Qualitative Study in Rural Yogyakarta Province, Indonesia”	To explore perspectives about hypertension from patients who do not take antihypertensive medications	Yogyakarta, Indonesia. Rural	30 participants—middle-aged and older adults	Qualitative description	Face to face semistructured interviews	Identified themes: (1) Alternative medicines for managing high blood pressure (2) Accessing healthcare services (3) The need for antihypertensive medications (4) Existing support and patients' expectations Reluctance to take antihypertensive medications was influenced by patients' beliefs in personal health threats and the effectiveness of antihypertensive medications, high self-efficacy for taking alternative medicines, the lack of recommendations regarding hypertension treatment, and barriers to accessing supplies of medicines.
Rahmawati and Bajorek [16]. “Access to Medicines for Hypertension: A Survey in Rural Yogyakarta Province, Indonesia”	To explore how and where people in rural villages in Indonesia obtain their supplies of antihypertensive medications	Yogyakarta, Indonesia. Rural	384 participants—male and female, middle-aged and older adults, high and low SES	Descriptive quantitative	Researcher-administered questionnaire	Among 384 participants, 203 people reported had taken medication for the latest 30 days before the data collection. 97 of 203 participants (50%) obtained hypertensive medications from public health services, while 61 participants (30%) get the medications from private healthcare providers (e.g., private hospital, community pharmacy, private nurse, private doctor, and private nurse), and 45 participants (22%) reported obtaining the medications from varied sources (e.g., pharmacy, community health centre)

**Table 4:** Descriptive summary of literature.

Descriptor	Studies *N* = 9
*Year of publication*	
2010	1
2016	1
2017	1
2018	3
2019	3
*Location*	
Urban	4
Rural	4
Urban and rural	1
*Gender*	
Females only	6
Males and females	1
Not specified	2
*Education*	
Educated	1
Less educated	5
Not specified	3
*Population studied*	
Middle-aged adults	2
Elderly	4
Middle-aged adults and elderly	1
Not specified	2
*Socioeconomic status*	
Low income	3
Middle income	1
Low and middle income	1
Not specified	4
*Medical diagnosis*	
Hypertension	3
Diabetes	4
Cardiovascular disease	1
End-stage renal disease	1
*Factors influencing self-care*	
Religion	3
Biomedical knowledge and health literacy	9
Cultural expectation	8
Family support	4
Peer support	2
Support from healthcare providers	4
Community support	3
Barriers to care	6
